# Supplementary material for: Dosimetric and biologic intercomparison between electron and proton FLASH beams
Source: bioRxiv. 2023 Apr 21:2023.04.20.537497. Preprint. [Version 1] doi: 10.1101/2023.04.20.537497 (PMC10153243; doi:10.1101/2023.04.20.537497)
Supplement: Supplement 1 [file NIHPP2023.04.20.537497v1-supplement-1.pdf]

Supplementary material

1.1 Animal irradiation setup at PSI-Gantry1

bioRxiv preprint doi: https://doi.org/10.1101/2023.04.20.537497; this version posted April 21, 2023. The copyright holder for this preprint (which was not certified by peer review) is the author/funder, who has granted bioRxiv a license to display the preprint in perpetuity. It is made available under aCC-BY 4.0 International license.

At PSI-Gantry1, a 250 MeV proton beam is transported from the cyclotron to the treatment nozzle with an efficiency of ~85%. In the nozzle, the pristine beam traverses 40 Polystyrene (PS) plates resulting in a residual energy of 170 MeV and a beam sigma of ~ 25 mm at 1 m distance downstream of the nozzle. A 6 cm thick copper collimator is used to shape the 17 mm large irradiation field. Additional shielding is placed adjacent to the collimator to protect the animals from any stray radiation. An illustrative view of the irradiation setup is provided in Figure 1.

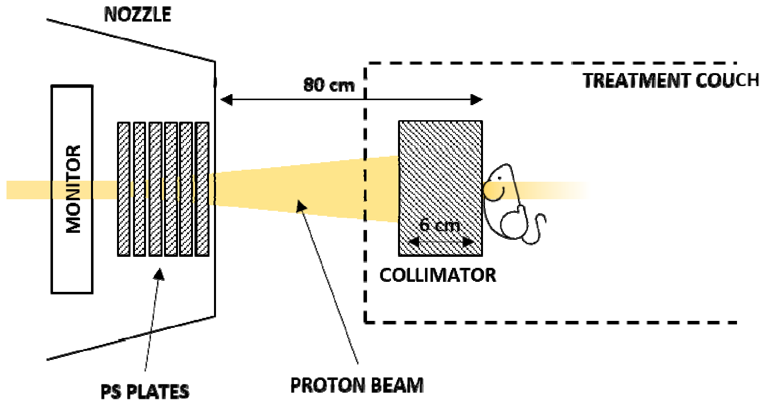

Supplementary Figure 1. Illustrative representation of the animal irradiation setup at PSI Gantry1 – view from the top (dimensions are not to scale).

The dose delivery is supervised by a monitor chamber *(22)* built in the Gantry nozzle. On the day of the experiment, the output of the machine is verified and eventually corrected by means of a commercial synthetic microDiamond detector (PTW, Freiburg, Germany) at the required dose rates. Additionally, a TM7862 transmission chamber (PTW, Freiburg, Germany) is used as a redundant monitor. The response of the TM7862 ion chamber is typically characterized beforehand against a Faraday cup *(25)*.

1.2 Phantom irradiation setup at PSI-Gantry1

Two of the PMMA phantoms loaded with passive detectors were irradiated at each dose rate, i.e. conventional (0.1 Gy/s) and FLASH (110 Gy/s) dose rate. The phantoms were positioned downstream of the copper collimator, aligned using a wall-mounted laser, with the rectangular face orthogonal to the beam axis (Figure 2). The same irradiation setup was replicated for the measurements with PSI reference dosimeters. The following elements were placed downstream of the collimator, in sequence and attached to each other: 11 mm thick PMMA slab, EBT3 film, microDiamond detector (Figure 3). The microDiamond was inserted into a plastic phantom to ease the positioning with respect to the collimator. Note that the microDiamond has an intrinsic water equivalent buildup of 1.1 mm *(38)* and a sensitive circular area with a 2.2 mm diameter.

bioRxiv preprint doi: https://doi.org/10.1101/2023.04.20.537497; this version posted April 21, 2023. The copyright holder for this preprint (which was not certified by peer review) is the author/funder, who has granted bioRxiv a license to display the preprint in perpetuity. It is made available under aCC-BY 4.0 International license.

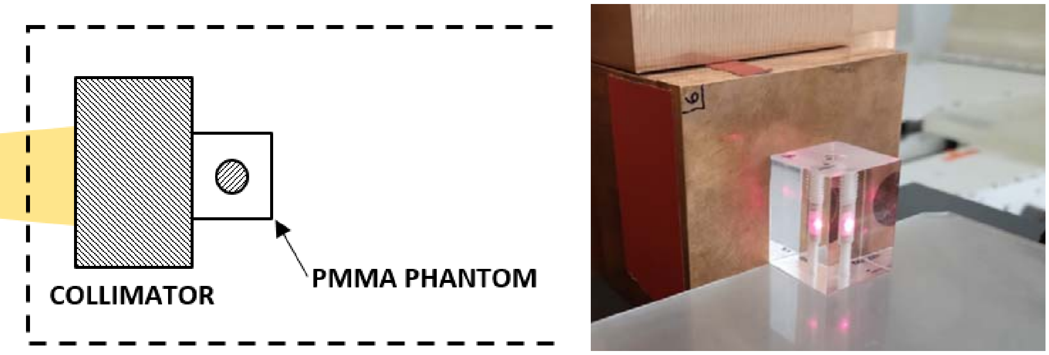

Supplementary Figure 2. Schematic diagram and picture of the irradiation setup for the PMMA phantoms.

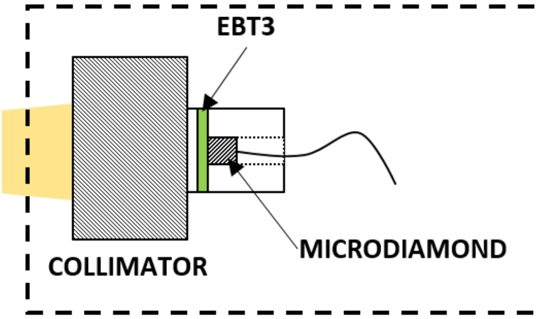

Supplementary Figure 3. Schematic diagram of the irradaition setup for EBT3 films and microDiamond.

The 2D dose distributions measured with EBT3 films – for the setup illustrated in Figure 3 – are shown in Figures 4 and 5 for conventional and ultra-high dose rate, respectively. Orthogonal beam profiles are shown as well.

The EBT3 dose reported in the Results section is the average dose – at the center of the field – evaluated over an area equal to the microDiamond sensitive area.

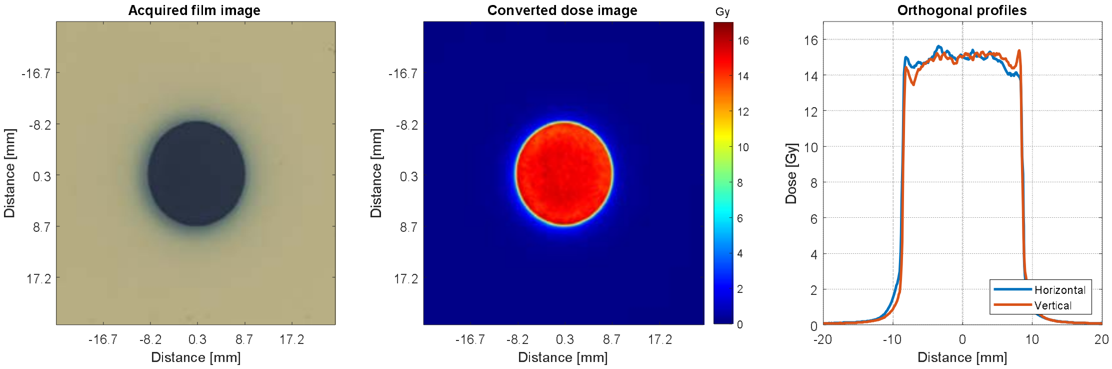

Supplementary Figure 4. EBT3 measured dose at conventional dose rate (0.1 Gy/s).

bioRxiv preprint doi: https://doi.org/10.1101/2023.04.20.537497; this version posted April 21, 2023. The copyright holder for this preprint (which was not certified by peer review) is the author/funder, who has granted bioRxiv a license to display the preprint in perpetuity. It is made available under aCC-BY 4.0 International license.

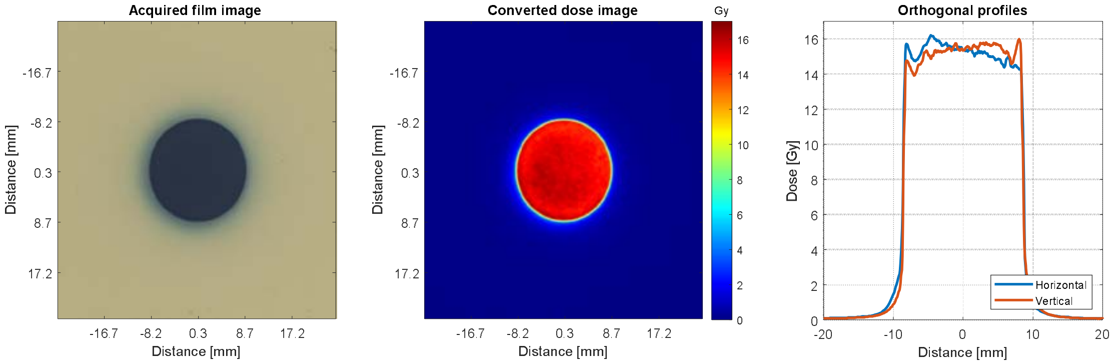

Supplementary Figure 5. EBT3 measured dose at FLASH dose rate (110 Gy/s).

1.3 Cross-calibration of phantom detectors at PSI-Gantry 2

We have performed proton irradiations at PSI-Gantry 2 to check the reproducibility of phantom detectors and to determine correction factors for the specific beam quality by cross-calibration against a reference ion-chamber traceable to METAS.

The phantom detectors (Alanine, TLDs and EBT3 films) were loaded into a dedicated holder that matches the dimensions of an Advanced Markus (PTW, Freiburg, Germany) ion-chamber. The holder can allocate up to 7 Alanine/TLD pellets and 14 laser-cut EBT3 films (Figure 6). The cap of the holder has the same water equivalent thickness as the Advanced Markus build-up cap. 7 x 3 times Alanine pellets, 7 x 3 times TLD pellets and 14 x 2 times EBT3 films have been irradiated. Before that, the dose was measured 3 times with the Advanced Markus ion-chamber. All detectors were irradiated at isocenter at a water-equivalent depth of 12.7 mm. Beam parameters were as follows:

- Initial energy 170 MeV – same energy as for the biological experiments at Gantry1
- Beam spots arranged in a grid with equal spacing (2.5 mm) and weights.
- Field dimensions 10 x 10 cm^2
- Field uniformity < 1%
- Field reproducibility < 0.1%

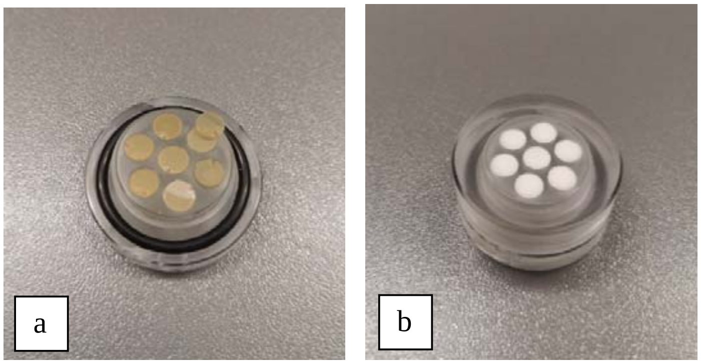

bioRxiv preprint doi: https://doi.org/10.1101/2023.04.20.537497; this version posted April 21, 2023. The copyright holder for this preprint (which was not certified by peer review) is the author/funder, who has granted bioRxiv a license to display the preprint in perpetuity. It is made available under aCC-BY 4.0 International license.

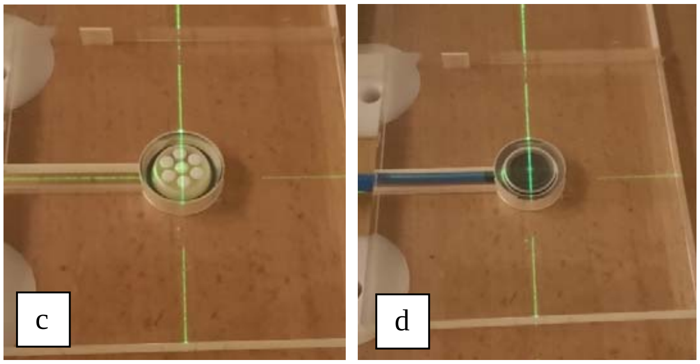

Supplementary Figure 6. Images of the dosimeters loaded into the dedicated holder: a) laser-cut EBT3 films, b) and c) Alanine pellets. The Advanced Markus (d) chamber was used as a reference detector.

In Figure 7, the dose measured by the different detectors is plotted for all subsequent irradiations. For Alanine, TLDs and EBT3 films, each point represents the mean value of the readings within a single irradiation, and the error bars represent the standard deviation of the detectors loaded into the holder. The number of detectors irradiated per delivery is reported on the x-axis. Additionally, the mean values over multiple irradiations and the related standard deviation of the mean are shown for each detector type. For the Advanced Markus, only a single data point is shown, being its reproducibility <0.3 %.
The measured reproducibility of the phantom detectors was 0.9 %, 3.7 % and 1.4 % for Alanine, TLDs and EBT3 films, respectively.

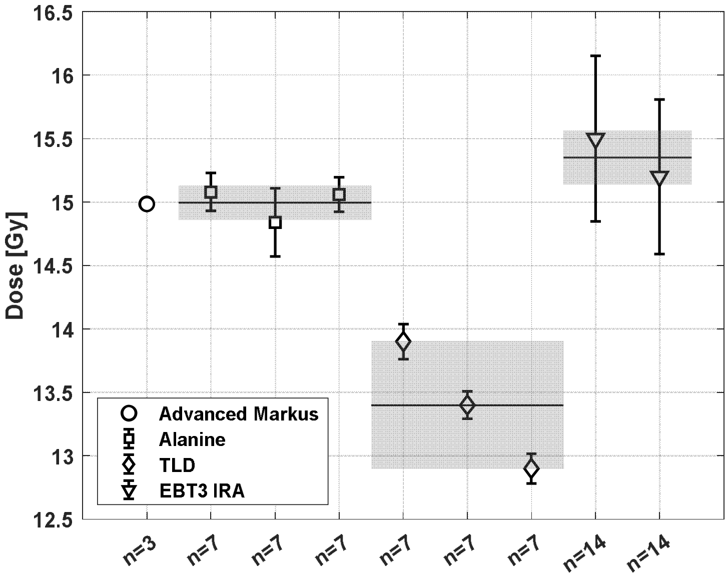

Supplementary Figure 7. Measured dose for repeated irradiations at PSI-Gantry 2. The number of detectors irradiated in each delivery is reported on the x-axis. The error bars represent the spread of the readings in each delivery (the Advanced Markus bars are smaller than the marker size). The horizontal black lines are the mean values, and the shaded areas are the standard deviation of the mean values of multiple deliveries for each detector type.

The dose measured by the Advanced Markus in the proton single-energy field can be expressed using IAEA TRS-398 formalism:

bioRxiv preprint doi: https://doi.org/10.1101/2023.04.20.537497; this version posted April 21, 2023. The copyright holder for this preprint (which was not certified by peer review) is the author/funder, who has granted bioRxiv a license to display the preprint in perpetuity. It is made available under aCC-BY 4.0 International license.

$$D_{w,Q}^{Adv.Markus} = M_Q^{Adv.Markus} N_{D,w,Q_0}^{Adv.Markus} k_{Q,Q_0}^{Adv.Markus} \quad (1)$$

where $M_Q^{Adv.Markus}$ is the reading of the chamber corrected for ambient and chamber specific parameters, $N_{D,w,Q_0}^{Adv.Markus}$ is the calibration factor determined at METAS in Co-60 reference beam, and $k_{Q,Q_0}^{Adv.Markus}$ is the beam quality correction factor for the chamber at the beam quality Q. Similarly, the dose measured by each of the phantom detectors in the same proton field can be written as:

$$D_{w,Q}^{detector} = M_Q^{detector} N_{D,w,Q_0}^{detector} k_{Q,Q_0}^{detector} \quad (2)$$

where the quantity $M_Q^{detector} N_{D,w,Q_0}^{detector}$ is provided directly by IRA after detectors readout and represents the absorbed dose to water in a Co-60 reference beam for the measured signal $M_Q^{detector}$.

Provided the irradiation field is the same for all detectors, it is possible to determine a beam quality correction factor for each of the phantom detectors by imposing

$$D_{w,Q}^{Adv.Markus} = D_{w,Q}^{detector}:$$

$$k_{Q,Q_0}^{detector} = \frac{M_Q^{Adv.Markus} N_{D,w,Q_0}^{Adv.Markus} k_{Q,Q_0}^{Adv.Markus}}{M_Q^{detector} N_{D,w,Q_0}^{detector}} \quad (3)$$

For this cross-calibration, we have chosen the same beam quality Q as used in the biological experiments at Gantry1, defined by an initial beam energy of 170 MeV and a measurement depth of 12.7 mm.

The beam quality correction factors were calculated according to equation (3) and found to be 1.00, 1.12 and 0.98 for Alanine, TLDs and EBT3 films, respectively. The associated uncertainties were estimated as in Palmans et al. study (including type-A uncertainties and uncertainties on $N_{D,w,Q_0}^{Adv.Markus}$ and$N_{D,w,Q_0}^{Detector}$) and amount to 2.3 %, 5.5 % and 2.5 %.
